# Short-Pulse Lasers: A Versatile Tool in Creating Novel Nano-/Micro-Structures and Compositional Analysis for Healthcare and Wellbeing Challenges

**DOI:** 10.3390/nano11030712

**Published:** 2021-03-12

**Authors:** Ahmed Al-Kattan, David Grojo, Christophe Drouet, Alexandros Mouskeftaras, Philippe Delaporte, Adrien Casanova, Jérôme D. Robin, Frédérique Magdinier, Patricia Alloncle, Catalin Constantinescu, Vincent Motto-Ros, Jörg Hermann

**Affiliations:** 1 Aix-Marseille University, CNRS, LP3 UMR 7341, Campus de Luminy, Case 917, CEDEX 09, 13288 Marseille, France; david.grojo@univ-amu.fr (D.G.); alexandros.mouskeftaras@univ-amu.fr (A.M.); philippe.delaporte@univ-amu.fr (P.D.); adrien.casanova@univ-amu.fr (A.C.); patricia.alloncle@univ-amu.fr (P.A.); catalin.constantinescu@univ-amu.fr (C.C.); jorg.hermann@univ-amu.fr (J.H.); 2 CIRIMAT, Université de Toulouse, UMR 5085 CNRS/Toulouse INP/UT3 Paul Sabatier, Ensiacet, 4 allée E. Monso, CEDEX 04, 31030 Toulouse, France; christophe.drouet@cirimat.fr; 3 Aix-Marseille University, INSERM, MMG, Marseille Medical Genetics, 13385 Marseille, France; jerome.ROBIN@univ-amu.fr (J.D.R.); Frederique.MAGDINIER@univ-amu.fr (F.M.); 4 Institut Lumière Matière UMR 5306, Université Lyon 1—CNRS, Université de Lyon, 69622 Villeurbanne, France; vincent.motto-ros@univ-lyon1.fr

**Keywords:** laser-assisted methods, nanoparticles, nanomedicine, laser printing, tissue engineering, laser machining, laser elemental analysis

## Abstract

Driven by flexibility, precision, repeatability and eco-friendliness, laser-based technologies have attracted great interest to engineer or to analyze materials in various fields including energy, environment, biology and medicine. A major advantage of laser processing relies on the ability to directly structure matter at different scales and to prepare novel materials with unique physical and chemical properties. It is also a contact-free approach that makes it possible to work in inert or reactive liquid or gaseous environment. This leads today to a unique opportunity for designing, fabricating and even analyzing novel complex bio-systems. To illustrate this potential, in this paper, we gather our recent research on four types of laser-based methods relevant for nano-/micro-scale applications. First, we present and discuss pulsed laser ablation in liquid, exploited today for synthetizing ultraclean “bare” nanoparticles attractive for medicine and tissue engineering applications. Second, we discuss robust methods for rapid surface and bulk machining (subtractive manufacturing) at different scales by laser ablation. Among them, the microsphere-assisted laser surface engineering is detailed for its appropriateness to design structured substrates with hierarchically periodic patterns at nano-/micro-scale without chemical treatments. Third, we address the laser-induced forward transfer, a technology based on direct laser printing, to transfer and assemble a multitude of materials (additive structuring), including biological moiety without alteration of functionality. Finally, the fourth method is about chemical analysis: we present the potential of laser-induced breakdown spectroscopy, providing a unique tool for contact-free and space-resolved elemental analysis of organic materials. Overall, we present and discuss the prospect and complementarity of emerging reliable laser technologies, to address challenges in materials’ preparation relevant for the development of innovative multi-scale and multi-material platforms for bio-applications.

## 1. Introduction

The healthcare and wellbeing challenges, including energy and environment correlated issues, that we face nowadays ask for an extreme anticipation and reactivity from the scientific community. Innovation and creation of functional materials, able to restore biological functions or to detect and treat diseases and infections, are of paramount importance [1,2,3]. The unsettled worldwide emergence of the severe acute respiratory syndrome coronavirus 2 (SARS-CoV-2) and the resulting COVID-19 pandemic, with its societal and economic consequences, is the perfect illustration [4,5]. In this context, important research efforts concentrate today on the use of the most advanced material engineering processes to elaborate functional platforms (e.g., nanostructures, substrates, scaffolds, etc.) with controlled physical, chemical and even biological properties toward healthcare and bio-applications [6,7,8,9,10].

A multitude of top-down and bottom-up nano-/micro-fabrication technologies have been developed with regards to engineered functional platforms [11]. In a first approach, the process is to reduce large bulk material down to range size thanks to mechanical (e.g., mechanical or ball milling) [12,13], ultrasonic (e.g., ultrasound bath) [14], or even thermal effects (e.g., plasma projection) [15]. However, such a process generally results in particles with a wide size distribution, an uncontrolled shape or contamination problems, making their potential use quite difficult, especially for biomedical applications. Wet chemistry, electrochemical and aerosol routes such as chemical vapor deposition and electrospray are other methods based on a bottom-up approach [16,17,18]. In most cases, these methods require to start from precursor species (e.g., salts, molecules, gas, etc.) in liquid or gas environments which lead to the formation of clusters of nanoparticles (NPs) by nucleation and coalescence, or other condensation phenomena. These processes lead to the creation of varieties of compositions, shapes and sizes of NPs, with remarkable physical, chemical and electronic properties [19]. For instance, due to their size-dependent interaction with light, a multitude of theranostic modalities including optical bioimaging, biosensing, light-activated therapy, thermal therapy and magnetic properties can be exploited [20,21,22,23,24]. Moreover, the size reduction of NPs down to values ranging from 1 to 100 nm confers furtiveness characteristics, leading to minimize immunity issues and precocity elimination. NPs structures (e.g., internal core or corona) can also be employed as a cargo-module for drug delivery while their ligand-free surface can allow coatings with varieties of (bio)molecules (e.g., DNA, antibody, etc.) and targeting ligands to enhance biodistribution and active dissemination toward the target cells, tissues or organs [25,26,27,28]. Despite several advantages, the major concerns about NPs are high agglomeration rates or undesired chemical coatings/residues due to the synthesis process. Moreover, usually, such techniques take place under complex physicochemical conditions (e.g., temperature, pH pressure, etc.), starting from hazardous reactants (e.g., citrate, HF, etc.), which can alter the interaction with the biological tissues and lead to potential contamination issues [29,30,31,32].

Other techniques, such as photolithography, soft lithography or electrospinning techniques, have been exploited in view of creating micro-/nano-hierarchical structures or substrates to closely emulate the complexity and functionality of the extracellular matrix and to re-create a mimicking environment that offers comparable control over cell activities [11,33,34,35,36]. In fact, tissues and organs have very complex and hierarchical structures from macro-/micro- down to nano-scale level, and many biological processes and interactions with the nanostructured extracellular matrix occur at the nanoscale level. The possibility to design hierarchical materials with superior physicochemical (e.g., mechanical, electrical, etc.) and structural properties (e.g., three-dimensional (3D) architecture, surface topography, porosity, etc.) plays a key role in healthcare applications (e.g., tissue engineering and regenerative medicine) [37,38]. Biocompatibility and biodegradability are other properties which have to be considered to limit any rejection or toxicity issues. Considerable efforts have thus been spent to elaborate suitable hierarchical substitute structures from extracellular matrix components (e.g., collagen, gelatin, etc.) as thin films or fibers to provide an adequate microenvironment to influence/control cells’ adhesion, migration and differentiation. Other techniques and strategies based on chemical or physical etching have been exploited to induce varieties of periodic texturing topographies of surfaces (e.g., nanowires, rods, cavities, etc.) in view of reproducing elemental topography cues that may control/regulate cell behaviors [39,40,41]. However, such processes are still complex (multiple steps, clean-room facilities, etc.), and require working in a controllable environment (e.g., toxic gas, metal catalyst, etc.), which may generate contamination issues.

Driven by the requirements of precision, repeatability, flexibility and cleanliness, short-pulse lasers appeared as multifaceted tools able to design a panel of engineered platforms, including ultraclean colloidal nanoparticles, micro-/nano-structured objects, 3D microenvironments, and even capable to perform multi-elemental analysis of bio-systems [42,43,44,45]. In fact, the interactions of short-pulse laser radiation with solid material lead to the ablation of ionized matter, which assemble to clusters of nanocrystals when the irradiation occurs in liquid environment (e.g., ultrapure aqueous solution) [46,47]. This process leads “naturally” to the formation of very stable and pure colloidal NPs suspensions without extra chemicals and toxic residues or waste [48,49,50]. Moreover, when the irradiation occurs in a low-pressure gas environment, such nanocrystal clusters can be deposited as a NPs-film coating on substrates [51,52]. By carefully adjusting the irradiation conditions (e.g., wavelength, fluence, repetition frequency), short-pulse lasers can serve as surface texturing tools to create varieties of micro- and nano-motives in view of tissue engineering applications [6,53,54,55,56,57]. In one single pulse, a short-pulsed laser can serve for high-resolution deposition and direct patterning with a variety of materials, including biological moiety without alteration issues to create a specific 3D microenvironment for bioprinting [58,59]. Finally, the plasma generated during irradiation beam can be exploited in the healthcare field to analyze clinical samples for trace elemental mapping or contaminants’ detection [60,61,62].

This article aims to review recent findings and perspectives of this research on the use of short-pulse lasers in healthcare and bio-applications. We report four illustrative examples including: (I) the pulsed laser ablation in liquids (PLAL), (II) direct laser writing, including microsphere-assisted laser surface engineering for surface structuring at nano-/micro-level, (III) laser-induced forward transfer (LIFT) as a high-resolution printing method and (IV) laser-induced breakdown spectroscopy (LIBS)-sensitive spatially resolved chemical analysis. We highlight their potential and complementarity in material science, towards the development and analysis of innovative platforms for healthcare applications.

## 2. Pulsed Laser Ablation in Liquids for Colloidal Nanoparticles Synthesis

### 2.1. Principle of PLAL Method

Emerged as a “green” synthesis method, pulsed laser ablation in liquid appeared as a relevant process in the elaboration of ultra-clean colloidal NPs for a variety of applications, including in electronics, energy production and nanomedicine [43,44,63]. In fact, the PLAL process exhibits numerous advantages over conventional chemical methods, such as the possibility of direct NPs synthesis in deionized water without stabilizing ligands or other organic dispersant molecules. Colloidal NPs formed under these conditions exhibit a unique “bare” (ligand-free) surface with high reactivity, making possible the functionalization with biomolecules (e.g., proteins, DNA, etc.) and organic functions (e.g., Amine, -COOH, etc.) in situ. Moreover, to condition an appropriate NPs physiochemical property including shape, size and stoichiometry, the liquid composition can be modulated by adding ionic species, or even replaced by organic solvents, oils and super-fluids [43].

It should be noted that short-pulse laser ablation relies on a complex interplay between various physical mechanisms depending on irradiation conditions. Several related questions remain a large subject of debate that strongly depend on the material and the laser regimes that are experimentally and theoretically investigated [43]. A detailed discussion on these fundamental aspects is out of the scope of this review paper concentrating on the laser potential for a specific range of applications.

However, we would like to highlight that laser ablation can be directly used for NPs preparation but tends to naturally deliver broad size particles distributions (from several tens to hundreds of nm) with polymodal size populations. As an accepted scenario, laser ablation is the result of extreme phenomena, such as shock waves, plasma plume expansion (cooling) and cavitation bubble explosion. It is established that the generation of NPs is the result of nucleation and coalescence of ionized species ejected from bulk material after absorption of incoming photons occurring during the ablation process. As a debated scenario, a sequence of different mechanisms occurs during plasma plume expansion (cooling) and the explosion of a cavitation bubble. Such phenomena are especially important for the “long” ns laser ablation regime. The addition of reactive chemical products during the ablation process makes efficient “chemical” control of NPs size possible, but in this case, the cleanness of NPs can be compromised. On the other hand, it is now accepted by the laser processing community that ultrafast regimes (using femtosecond pulses) give access to the highest level of controllability. The ultrafast character of interaction means that much less radiation is transferred to the cavitation bubble, which limits cavitation phenomena and thus makes much more rigorous control of NPs size and size dispersion possible [43].

**Figure 1:**
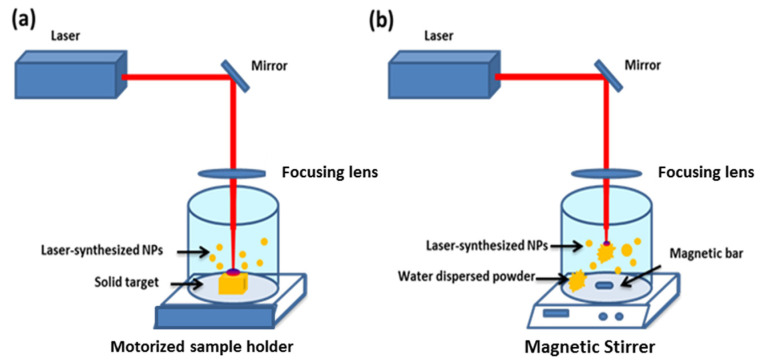
Principle of laser-based nanoparticles (NPs) synthesis via successive steps of (**a**) generation through ablation and (**b**) size-tuning via fragmentation.

In a conventional ablation protocol (Figure 1a), the experimental set-up consists of a pulsed laser beam focused onto a solid target surface [64]. To promote optimal surface ablation, the target is constantly moved with a motorized sample holder [50,65,66]. As an alternative set-up, the ablation protocol can be switched to a fragmentation configuration when the ablated material is a water dispersed powder (Figure 1b) [64,67]. In this case, the pulsed laser beam is directly focused inside the synthesis medium and the solution is continuously stirred to ensure a homogenous fragmentation process. Finally, the combination of both is a very efficient way to obtain a desired size with a very narrow size distribution of formed NPs. Since laser ablation occurs in a very short time (a few nanoseconds) and under extreme physical conditions (thousands of kelvins and hundreds of megapascals), many experimental methods (e.g., spectroscopic and acoustic measurements, CCD (charged coupled device) cameras’ observations, etc.) and theoretical modelling investigations were developed to describe and understand this process [68,69]. The fragmentation mechanism remains only partially understood. However, “photothermal evaporation” and “Coulomb explosion” are reported as the main mechanisms responsible for ablation depending on situations. Moreover, this fragmentation approach is the basis of other NPs preparation methods, starting from micro-powder suspensions instead of a substrate.

### 2.2. Generation of Laser-Synthesized Colloidal NPs

As mentioned above, one of the main advantages of PLAL over chemical methods is the possibility to generate colloidal NPs formulations without surfactant agents or other stabilizing molecules [43]. Moreover, by adjusting laser parameters (e.g., focusing point, laser-beam duration, repetition rate, etc.) and physicochemical conditions (e.g., pH, temperature, solvent, ionic strength), it is possible to elaborate a variety of inorganic and metallic NPs and their derived formulations [43,50,70,71,72,73].

**Figure 2:**
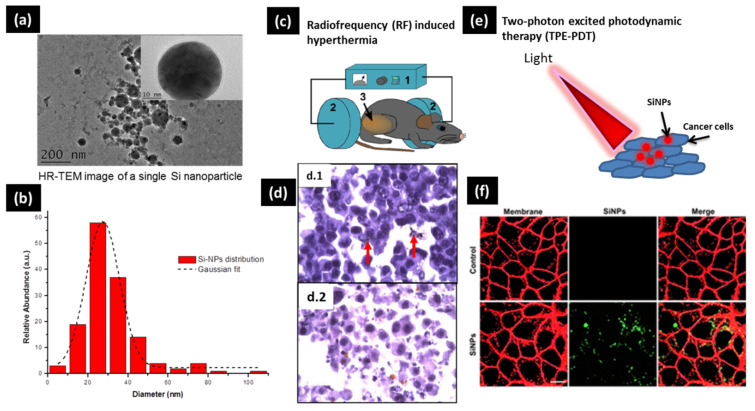
(**a**) Typical HR-TEM (High-resolution transmission electron microscopy) image of SiNPs produced by pulsed laser ablation in liquid (PLAL) with corresponding diameter size distribution (**b**). (**c**) Schematics of the RF (Radio frequency) radiation-based therapy setup with inhibition tumor effect after 1 h (d.1) and 3 days (d.2) of RF-based treatment using SiNPs as nano-sensitizers (**d**). (**e**) A schematic drawing of the principle of two-photon excited photodynamic therapy. (**f**) Confocal microscopy imaging of inhibition effect of SiNPs on living cells MCF-7 breast cancer after two-photon excited photodynamic therapy treatment at 900 nm. (**a**) and (**b**) are adapted from Reference [83], (**c**) and (**d**) are adapted from Reference [81], (**e**) and (**f**) are adapted from Reference [82].

For instance, based on the fragmentation geometry, it have shown the possibility to elaborate ultraclean and very stable (over more than six months) colloidal silicon nanoparticles (SiNPs) for biomedical applications by focusing ultrashort femtosecond laser pulses into pure water [67,72,73]. In fact, beside its great interest in the field of microelectronics, silicon is a bioactive element of interest for many physiological processes such as bone mineralization [74,75,76]. Moreover, based on its intrinsic physicochemical properties, SiNPs offer a panel of theranostic modalities in imaging (e.g., photoluminescence-based imaging, etc.) and therapy (e.g., hyperthermia therapies, photodynamic, etc.) [77,78,79,80,81,82]. Due to the presence of a thin oxidized surface layer (SiOx^–^ with 0 ≤ x ≤ 2) with a ζ potential of –40 ± 2.01 mV, SiNPs offer efficient interactions with biological materials (e.g., proteins, DNA) and chemical functions (e.g., Amine, -COOH). We have elaborated bare colloidal SiNPs with controllable mean size in the range of 10–100 nm with a narrow size dispersion (Figure 2a,b) [72,83]. Moreover, by playing on the dissolved oxygen rate in the synthesis medium, we demonstrated the possibility to control the dissolution rate on demand [83]. Based on systematic in vitro and in vivo tests, SiNPs were used as relevant sensitizers for radiofrequency-induced hyperthermia with efficient tumor inhibition at relatively low concentration and with a minimum toxicity (Figure 2c,d) [81]. Very recently, starting from a Si wafer target, SiNPs were also investigated in a two-photon excited photodynamic therapy at 900 nm, with a promising cell death of 45% in case of MCF-7 (Michigan cancer foundation-7) breast cancer cells, as a consequence of intracellular reactive oxygen species release (Figure 2e) [82]. Moreover, their luminescence emission inside the cells was clearly observed in the ultraviolet-visible domain (Figure 2f).

**Figure 3:**
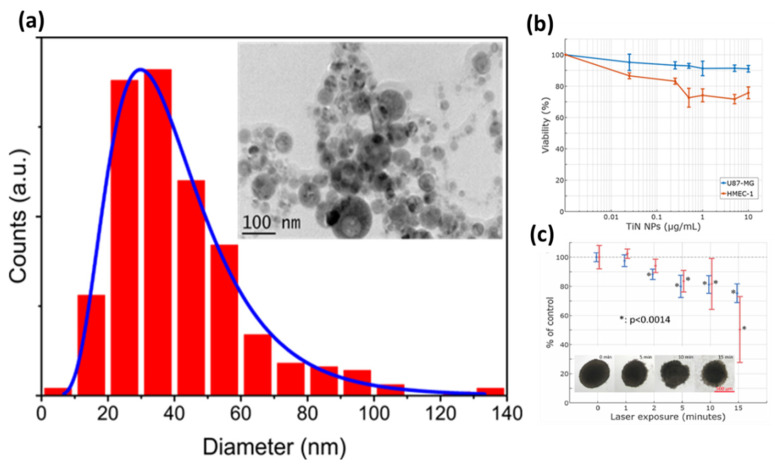
(**a**) TiNNPs synthesized by pulsed laser ablation in acetone at 100 μJ pulse energy with corresponding size distribution. (**b**) Viability of HMEC (Human mammary epithelial cells)(red line) and U87-MG (blue line) cells as functions of TiNNPs concentration. (**c**) Photothermal effect of TiNNPs on U87-MG cells at different laser exposure times. (**a**), (**b**) and (**c**) are adapted from Reference [85].

Thanks to their unique physical properties associated with the collective oscillations of free electrons known as surface plasmon resonance, AuNPs have also attracted great interest. Thus, a laser-synthesized AuNPs formulation was developed, and first, biological studies including in vitro and in vivo tests were achieved, opening up exciting perspectives of applications in nanomedicine [50]. Moreover, we have also shown the possibility to decorate “on demand” SiNPs by small AuNPs linked all together by organosilane APTMS (3-Aminopropyl)trimethoxysilane molecules and then assembled into complex core-satellite structures. We demonstrate that such platform makes possible the generation of a plasmonic absorption feature in the tissue transparency window, even for relatively small sizes of core-satellite constituents (less than 40 nm) [84]. We have also elaborated promising alternative plasmonic NPs with the TiN composition for potential photothermal therapy modalities (Figure 3a) [85]. In fact, exhibiting red-shift absorption/scattering characteristics toward the near infrared spectral range, TiNNPs can prove to be relevant tools for therapy tasks, especially in the relative tissue transparency region [85,86,87,88,89]. Based on the femtosecond laser ablation approach, we have shown the possibility to obtain ligand-free TiNNPs of 30 ± 2.01 nm size, with a great stability over six months. Thanks to a strong and broad plasmonic effect in the range of 640–700 nm, we demonstrated a strong photothermal therapeutic effect on U87-MG (Uppsala 87 malignant glioma) cells based on an in vitro three-dimensional (3D) model without significant toxicity (Figure 3b,c) [83].

**Figure 4:**
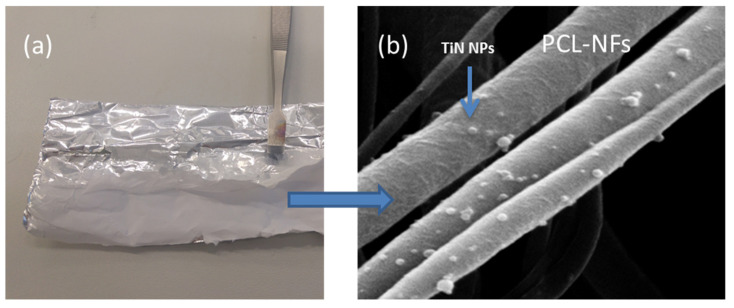
Illustrative image of polycaprolactone nanofibers decorated with TiNNPs elaborated by the PLAL process (**a**) with its corresponding SEM (Scanning electron microscopy) micrograph (**b**). (**b**) is adapted from Reference [95].

Besides potential applications in medicine, we have also explored laser-synthesized NPs as (multi)functional additives toward tissue engineering [64,90,91,92]. In fact, nowadays, conventional scaffolds made from ceramics, polymers, hydrogels and composites still suffer from several limitations, such as structural instability in biological fluid, poor bioactivity and primitive treatment modalities (e.g., unmonitored drug release with potential side effect/drug resistance) [93,94]. The possibility to associate functional/bioactive NPs to scaffolds can improve intrinsic properties (e.g., mechanical, electrical) and confer advanced biomedical/biological properties. It is expected that the reactive NPs surface, free from any ligands, offers a great reactivity toward the biological matrix. This approach is already comforted by first tests conducted on hybrid electrospun chitosan-polyethylene oxide (PEO) nanofibers. In this research, nanofibers functionalization was reached thanks to the electrostatic interaction between the polycationic nanofibers’ surface and the oxidized NPs surface. Thus, first benefits were shown, such as fiber diameter reduction and higher thermal stability, suggesting new bioactive performance and sensitivity toward temperature which can be exploited for therapeutic applications, as in hyperthermia for example. Another electrospun-polymer formulation based on polycaprolactone is now explored in combination with plasmonic NPs (e.g., TiNNPs). In fact, in addition to its biocompatibility and biodegradability properties, such polymers exhibit great stability in wet medium without any post-stabilization treatment (e.g., neutralization), which permits a long biological study and promising developments of smart scaffolds for tissue engineering aiming theranostic applications (Figure 4a,b) [95].

## 3. Direct and Microsphere-Assisted Laser Methods for Material Nano-/Micro-Engineering

Emerging direct laser writing technologies appear as particularly relevant solutions for multi-scale periodic material structuring and therefore engineering of bio-templates. Using lasers, structuring becomes flexible and the structure designs (potentially inspired by nature) can be optimized for cellular adhesion/compatibility or, on the contrary, for cell-repellent or antiseptic aims on medical devices and implants. Here, we concentrate on flexible digital processing methods. We describe two approaches for surface machining: Bessel-beam and microsphere-assisted focusing methods, because they ideally complement each other to address structuring at different scales down to nanometer dimensions. We also discuss femtosecond laser 3D writing as an approach able to add anywhere inside a transparent materials optics, mechanics of fluidics functionalities, an aspect particularly relevant for lab-on-chip bio-applications.

### 3.1. Precision Material Surface Machining with Femtosecond Bessel Beams

Short-pulse laser interactions allow localized energy deposition in materials that are at the basis of the laser nano-/micro-structuring technologies. Sub-micrometer precision in machining today is routinely achieved. The advent of femtosecond lasers has been a particular breakthrough on the process controllability because (i) the ultrafast character of interaction leads to limited thermally affected surrounding zones and (ii) the nonlinear energy deposition of intense pulses induces a strict threshold material-response that permits to confine the effects on dimensions smaller than the apparent spot size [42,96,97,98]. The latter also adds broad-spectrum material usability, including transparent solid materials and biological substances [98]. This has introduced the important field of laser nano-surgery of cells and tissues [99].

Despite these performances, practical optical limits remain when high-resolution processing is targeted, and even more so when uneven substances such as biological tissues or bio-compatible/bioengineered materials have to be processed. In such cases, conventional methods exploit tightly focused beams that limit the depth of focus (defined by the Rayleigh range), corresponding to the range of distances the target sample can be moved away from best focus without degrading the resolution. For instance, with simple estimates from Gaussian beam optics, the Rayleigh range depth becomes on the order of the spot diameter of micrometer dimension created with a near-infrared laser. This imposes severe positioning difficulties and makes it hardly possible to achieve reliable long-range sub-micrometer precision material processing with diffraction-limited Gaussian beams.

To solve this and some other difficulties, today, there are various advanced beam-shaping solutions available to deviate from conventional Gaussian or so-called “top-hat” intensity profiles [100]. In this way, complex patterns can be written directly, and parallelization can be implemented to reduce processing times and enable high-throughput production. Among the various beam shapes already demonstrated, Bessel-like beams attract particular interest because this approach solves the conflicts between resolution and depth of focus discussed above [101]. The ideal Bessel beam has a profile described by a Bessel function of the first kind that is a non-diffractive solution of the electromagnetic wave equation. This means that it does not diffract and diverge as it propagates. Such features make it possible to access subwavelength resolution while suppressing the need for precise positioning of the target on the optical axis [102,103].

**Figure 5:**
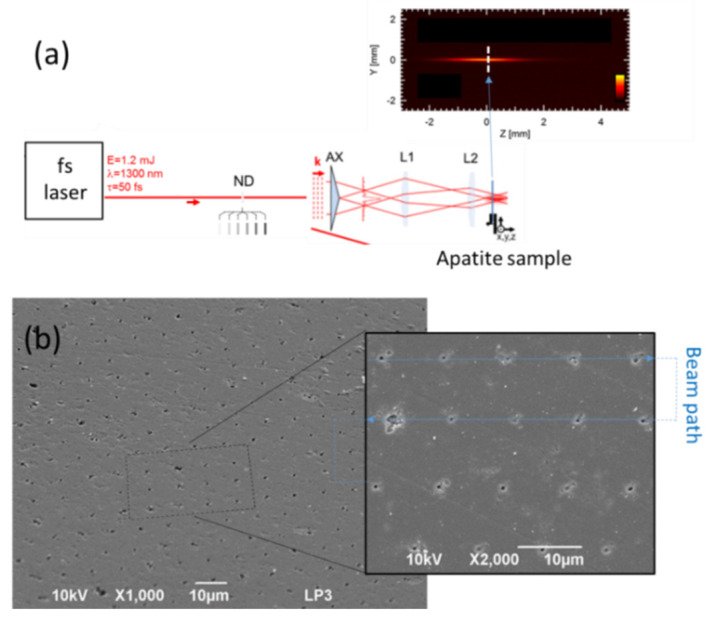
Periodically micro-structured stoichiometric sintered hydroxyapatite surface by direct femtosecond laser ablation. (**a**) Experimental arrangement for localizing laser radiation on a sample surface with micrometer size resolution over an extended depth of field. This allows to rapidly scan a large area of the sample with the beam to directly produce an array of identical craters. AX is a low-angle axicon producing a Bessel laser filament miniaturized with a 4-f imaging system provided by two lenses: L1 and L2. The inserted top image is a plasma luminescence in air evidencing the delivery of a high-intensity small filament extending over millimeters along the optical axis. (**b**) SEM images (tilted general view and zoomed top-view) illustrate the level of control that can be obtained. The periodicity is controlled with the scan speed and femtosecond laser repetition rate.

In practice, approximations of Bessel beams can be made with relatively simple arrangement of passive optics. A key is the use of a conical lens (so-called axicon) to generate a Bessel–Gauss beam [104]. In our arrangement shown in Figure 5a, a small-angle axicon (5° base angle) is used and lenses reduce the Bessel spot on target. According to the 4-f arrangement used here, a demagnification factor of f1/f2 = 1/8 is obtained with the focal lengths f1 = 25 mm and f2 = 200 mm for the lenses L1 and L2, respectively. Delivering a beam with a mJ-class femtosecond laser in this setup [105], one exceeds the optical breakdown threshold in air (intensity I > 10^14^ W/cm^2^) and the plasma emission evidences the produced laser filaments. The accompanying image (see insert in Figure 5) evidences the localization of the laser energy on a spot of micrometer dimension with a depth of field exceeding 1 mm, a performance totally inaccessible with conventional Gaussian beams. This allows to process large-scale surfaces by rapid scanning of the sample (or beam) without the need for precise positioning. We illustrate the level of performance with Figure 5b showing a calcium phosphate apatite pellet processed for periodic structuring. We note sub-micrometer ablation sites created by repeated single-pulse irradiations synchronized with sample motion. As confirmed by similar experiments conducted on other ‘pure’ materials (crystals) [105,106], the defects of repeatability that can be observed are based on sample inhomogeneities and/or damage precursor defects inherent to the biomaterial. Biomimetic calcium phosphate apatite is well-known for its chemical and crystallographic resemblance to the mineral bone tissue. Moreover, its intrinsic biocompatibility and the nanometer dimensions of its constitutive crystals not only allow one to envision applications in bone tissue regeneration, but also in other medical fields such as nanomedicine, as we have shown [107,108,109,110]. Since surface topography directly affects cell behavior, the possibility to elaborate controlled and calibrated architecture surfaces at different scale levels without any specific chemical treatments is particularly relevant for the design of bone substitute materials and could bring physical factors to apatite-based biomaterials bioactivity, for example in link with the bio-integration of the implants [6,54,55].

### 3.2. Microsphere-Assisted Sub-Diffraction Laser Processing

To achieve structuring at higher resolutions (100 nm and less), one needs to deviate from propagative optics and turn to evanescent near-field responses so that diffraction optical limits do not apply for energy deposition. In comparison to previously discussed cases, it is obviously at the expense of being bound to the vicinity of a specifically prepared structure. Two main ways of highly confined laser fields can be created. The first one is the laser coupling to the charge density oscillations in small-scale metals with plasmonic effects [111]. Nevertheless, absorption and the associated spectral resonance effects often constitutes an obstacle for prospective applications in the context of intense laser processing. A strong motivation thus exists in applying near-field solutions mediated by transparent dielectric structures. The concept of photonic nano-jets originating from the irradiation of dielectric microspheres is particularly relevant for that purpose. By definition, a photonic nano-jet is an extremely narrow local light field that propagates over several optical wavelengths while maintaining a sub-wavelength full width (or diameter) at half-maximum. This near-field phenomenon with more or less contribution from evanescent fields is easily observed with the focusing of the incident plane waves by relatively large transparent spheres (1–100 µm) [112]. Interestingly, recent advances have demonstrated that this attractive feature is also accessible with sub-micrometer spheres provided that a specifically engineered core-shell architecture is implemented [102]. An important consequence is that a monolayer of polystyrene or dielectric spheres (transparent) can serve as a micro-/nano-lens array for direct periodic illumination of a surface by nanobeams. The processing resolution is then defined by the focusing power of the spheres and the periodicity is controlled by the sphere diameters used to prepare the monolayer.

**Figure 6:**
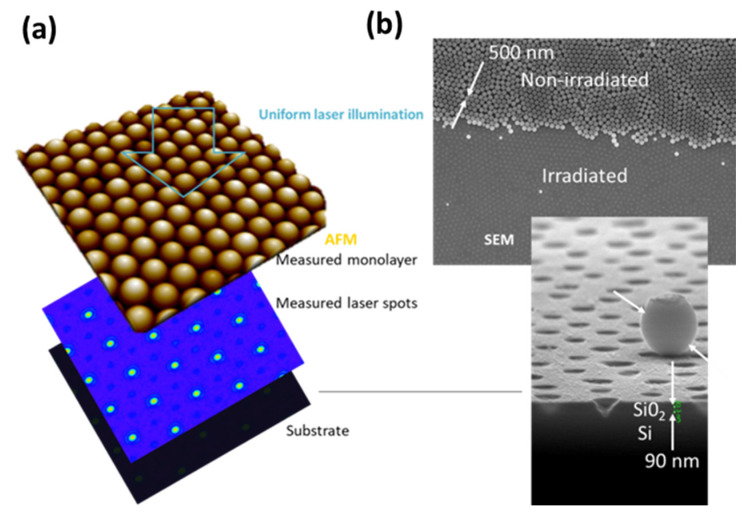
Porous membrane prepared by microsphere-assisted laser ablation. (**a**) Sketch illustrating the methodology in which a monolayer of dielectric microsphere is prepared (atomic force microscopy (AFM) image) to produce an array of near-field small spots, as shown by optical microscopy with top-illumination at a 400 nm wavelength in the plane supporting the monolayer. (**b**) Oxidized silicon surfaces supporting a monolayer of silica spheres (500 nm diameter) and partially irradiated with a nanosecond ultraviolet (UV) laser for periodic ablation. The SEM tilted view shows that the 90 nm oxide layer is perforated, leading to the formation of a porous membrane.

**Figure 7:**
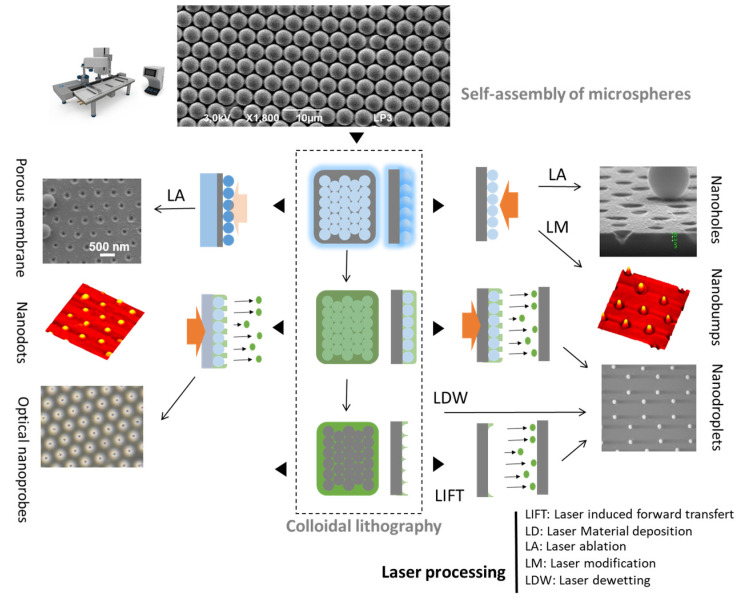
Nanofabrication by microsphere-laser joint methods. Most of the technologies in the panel of laser processing methods, that are additive, subtractive or corrective, can benefit from the focusing power of microspheres for large-scale synthesis of periodic structures at submicron scales. Inserted final structure images are extracted from references, all mentioned in the text.

This represents the basis of a large range of particle-assisted laser processing technologies that can be combined with other material preparation methods for obtaining of various types of nano-architectured templates. The general method is illustrated by the Figure 6 and Figure 7. A key aspect, in common with so-called colloidal lithography, is the preparation of self-assembled arrays of spheres [113], that is a material science topic attracting special attention. In this context, the Langmuir-Blodgett technique is a widely applied method due to its ability to prepare large-scale high-quality self-assembled monolayers, provided that the spheres and/or the surface are appropriately functionalized [114]. Figure 6a shows, by atomic force microscopy (AFM), the hexagonal periodic arrangement of functionalized spheres (1 µm diameter) as obtained by this method. For studying the focusing power of the spheres, one can then rely on optical microscopy techniques [115,116]. An illustration is given on the same figure, where the measurement of the small laser spots underneath illuminated spheres with 400 nm wavelength is provided. Here, it is important to highlight that this image tends to describe the laser energy distribution at the interface between the monolayer and the surface supporting the spheres. However, a drawback with this far-field diagnostic is that the images do not account for potential evanescent field contributions. This is solved experimentally by other indirect methods in which the spheres are in contact with photo-sensitive materials or materials that can be damaged when irradiated, so that the near-field energy distribution can be imprinted on the materials and subsequently observed by ex-situ high-resolution material imaging methods (e.g., SEM or AFM) [117].

For monolayers irradiated with single laser pulses with conditions exceeding the material ablation threshold on each spot, the particles are ejected with the ablated products by a laser cleaning process and leave an array of nanoholes directly behind on the surface that is directly attractive for optical sensing or structuring applications [118,119,120,121]. This process is illustrated with Figure 6b, where the top SEM image shows an array of 500 nm diameter spheres partially irradiated with a nanosecond laser at a 193 nm wavelength. The monolayer is supported by a silicon sample with a 90 nm oxide layer. As confirmed by the SEM side view, the laser process creates a nano-porous oxide membrane with size parameters (pore, periodicity) that can be varied with the sphere characteristics [122]. Porous membranes are potentially directly relevant for medical and bio-applications, but they also serve as deposition masks for the preparation of more complex nanomaterials, such as nanodot or rod arrays, after dissolution of the membrane [123,124]. More generally, the advantage is the inherent high resolution provided by the focusing power of the microspheres that can be used in almost all laser processing approaches, including laser de-wetting [125], machining [126,127], annealing and printing by the so-called laser-induced forward transfer [125,128], a technology detailed in Section 4. Combined with lithographic and other standard material preparation techniques, this leads to very cost-effective and flexible methods for the synthesis of various periodically architectured materials, as illustrated with Figure 7.

### 3.3. Femtosecond Laser-Induced Bulk Modification in Transparent Materials for Biological Applications

Complementing laser surface engineering methods to produce 2D (dimensional) or 2.5D structures, laser-induced bulk modification in transparent materials offers the possibility to add a large panel of new functionalities in the three dimensions of materials. This is the basis of lab-on-chip systems that open panels of biological and biomedical applications. The peak intensity of the beam, defined as the ratio between the fluence and the pulse duration, obviously reaches extreme values with femtosecond pulses of even modest energies. This is the basis of precision 3D writing inside transparent materials because appropriate laser beam focusing allows, by nonlinear energy deposition, confined and localized material breakdown in the focal region that can be positioned anywhere in the 3D space inside any transparent materials. In this way, laser writing opens the opportunity for fully integrated, 3D microsystems.

Processing materials by ultrashort laser pulses may give rise to mainly three types of modifications depending on pulse energy and pulse duration [129]: material densification, nanograting writing and micro-explosion. The first regime may be exploited for waveguide writing inside the bulk of the material. The second regime can be exploited in laser-assisted chemical etching and the third regime corresponds to material ablation. Using a commercially available femtosecond laser (~300 fs) and appropriately choosing the pulse energy, it is possible to switch between three regimes.

An emerging field, where femtosecond laser processing has been of great importance, is that of lab-on-chip applications [130]. As an example, using subtractive processing, it is possible to fabricate microfluidic networks inside a transparent material, and by switching to the waveguide writing regime, we can combine these two on a miniaturized laboratory used for detecting specific substances. The advantages of scale reduction of the biochips are numerous, and they include extreme compactness, ease of fabrication, low reagent consumption, high sensitivity in detection and low waste production. The fabrication steps and examples of realization are addressed below.

There are two fabrication processes that allow subtractive processing in transparent materials (mainly glasses): femtosecond laser-assisted etching [131] and water-assisted femtosecond laser drilling [132]. In femtosecond laser-assisted etching, either photosensitive (Foturan glass) or common fused-silica glass can be used as the host material. While the operational principle is similar—laser-exposed material has higher etching rate—the choice of the host material will slightly modify the process and the properties of the modification. In the case of photosensitive glasses, first, a critical amount of laser energy above the photoreaction threshold is deposited inside the medium. Followed by a first thermal treatment, crystal clusters are formed with a 50:1 etching ratio (defined as the laser-exposed to unexposed material) in diluted HF solution. Then, chemical etching is performed in HF to remove the exposed volumes and a final post-thermal annealing step ensures smoothening of the glass. An example of realization using that process is a nano-aquarium used for 3D observation flagellum motion of *Euglena gracilis* [133] and a microfluidic configuration for studying the gliding mechanism of *Phormidium* in soil for vegetables’ growth acceleration [134].

As mentioned before, simple fused-silica glass can also be used as the host material for biochips. The process is considerably simplified as no thermal treatment step is needed. Indeed, the increased etching rate of the exposed regions is mainly due to reorganization of the glass matrix (densification) and defect generation [135]. This process has been used for fabricating a 3D mammalian separator [136], the separability being based on the combination of T-junction configurations and microchannels with narrow diameter, allowing only deformable cells to pass through the channels. Using the same method, a monolithic cell counter based on 3D hydrodynamic focusing has been realized [137]. Using the same technology, waveguides were inscribed inside the host material which allowed reaching counting rates up to 5000 cells/s.

Even simpler is the subtractive processing using water-assisted femtosecond laser drilling. In this case, the glass sample is immersed inside distilled water and the laser is switched to the ablation regime. In the presence of water, the ablation debris are removed and micro- to nano-metric diameter channels can be written [138,139]. Furthermore, using this method, tapering of the microfluidic channels is avoided. As in dry ablation, the length of the ablated channel is rapidly limited by debris clogging. This can be surpassed by applying water-assisted femtosecond laser drilling in mesoporous glass followed by annealing [140]. The pores ensure better water delivery to the ablated region while annealing induces the collapse of pores, obtaining a highly transparent glass with the previously written structures always present. Channels with total length of the order of a centimeter and diameter of the order of 10 μm were written using this technique in order to achieve three-dimensional microfluidic mixing [141].

**Figure 8:**
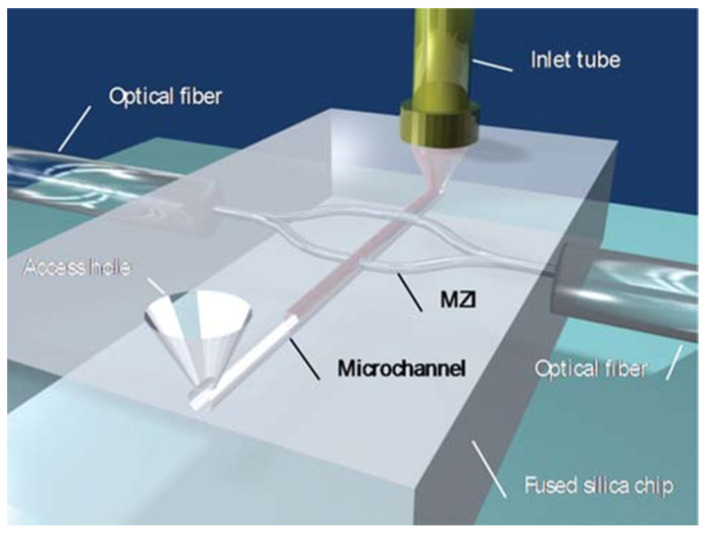
Schematic of the femtosecond laser-fabricated microfluidic channel and integrated Mach-Zender interferometer. The sensing arm crosses the channel orthogonally, while the reference one passes over it. Adapted from Reference [142].

By combining subtractive to additive processing, it is possible to reach a higher level of function integration in biochips. Thus, it is possible to not only machine a microfluidic channel network but also to inscribe, for example, waveguides to allow precise detection and analysis of the flows. An example of a detection scheme is the laser-inscription of a Mach-Zender interferometer, where the species concentration in a flow translates directly into phase shift and thus becomes measurable (Figure 8). Such a monolithic optofluidic device has been used to measure glucose with a sensitivity of 10^−4^ RIU (Refractive index unit) and a detection limit of 4 mM [142]. Another example of hybrid processing is using optical guidance inside a microfluidic waveguide to allow detection of very low concentrations of glucose (down to 200 nM) [143]. The host material is a photo-structurable glass, where first a microfluidic channel is produced by subtractive femtosecond laser processing and then a waveguide is inscribed to guide light into the liquid-filled microchannel. Coating the microchannel with a polymer ensures that the guiding conditions are met, and different concentrations of glucose translate as different optical densities that can be directly measured. Another application of femtosecond laser hybrid manufacturing concerned the detection of single red blood cells in diluted human blood inside a manufactured microchannel by two optical schemes [144]. The first involved sensing the intensity change of waveguide-delivered He–Ne laser light (632.8 nm) induced by the refractive index difference of a cell flowing in the channel. The other approach was via detection of fluorescence emission from dyed red blood cells excited by Ar laser light (488 nm) delivered by the optical waveguide. The lab-on-chip hybrid system, fabricated exclusively by femtosecond laser processing, is an extremely promising technique. In the future, novel femtosecond laser-inscribed functionalities, including electrical, optical and mechanical components, shall open up the door for unprecedented monolithic integration of biochips.

## 4. Laser-Induced Forward Transfer (LIFT)

### 4.1. Laser-Induced Forward Transfer for Biomedical Applications: Bio-Printing Approach

Printing techniques applied to biology make it possible to print either biocompatible inert material structured to accommodate cells or tissues, or living material, the latter application being called “bio-printing” [145,146,147]. These techniques began to be developed in the early 2000s and have undergone major optimization in recent years, including in industry. Their main goal is to deposit point-by-point a combination of biological and chemical elements that constitutes the extracellular matrix together with cells. Their applications range from tissue engineering for organ creation to regenerative medicine [148,149,150,151] and the discovery of new drugs [152]. These techniques open new perspectives for therapeutic purposes, allowing the creation of 2D or 3D in vitro bio-models strongly mimicking the in vivo environment, which could represent promising and powerful tools for the study and better understanding of rare pathologies and the discovery of new therapeutics for screening purposes techniques or tissue modelling, for example.

Various techniques are being considered, such as contact printing, inkjet or extrusion [153]. The development of bioprinting applications has led to the emergence of several companies that operate under three main business models [154]: manufacturing and sale of bioprinters, sale of bioprinting services, or partnership with a customer on a specific application. They give good results in terms of printing reproducibility but suffer from some limitations. For inkjet printing, if the obtained resolution is high, it is impossible to use bio-inks with high viscosity or containing a high cell density, which leads to nozzle clogging. Regarding extrusion, if the process is compatible with high viscosity and high cell density, the obtained resolution is lower.

**Figure 9:**
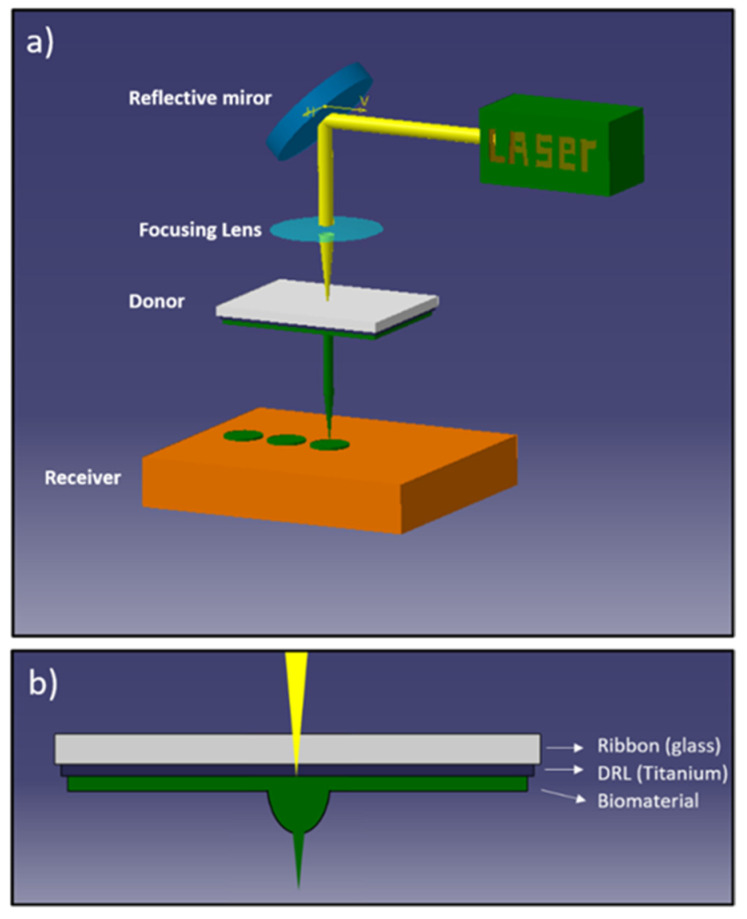
(**a**) Sketch of laser-assisted bioprinting technique: the laser pulse is focused by a lens on the donor substrate surface. The laser–matter interaction induces the ejection of the targeted material and its deposition on the receiver substrate in close proximity. (**b**) Zoom on the donor substrate during biomaterial ejection: the laser–matter interaction with the absorbing dynamic release layer (labelled DRL) creates an expanding and highly confined plasma, which leads to the generation of a hemispherical cavitation bubble which pushes the biomaterial layer away from the donor substrate [157].

Laser-assisted printing provides solutions to the above-mentioned limitations. Laser-Induced Forward Transfer was introduced by Bohandy in 1988 on copper printing [155]. As shown in Figure 9, the LIFT process consists of using a laser pulse to irradiate a thin film (material to be printed), which was previously deposited on a substrate transparent to the laser radiation. This coupled substrate/thin film is called “donor substrate” and its irradiation by the laser pulse leads to laser–material interaction processes that occur at the interface and lead to the ejection of a small portion of the targeted material and its deposition on a second substrate called “receiver” placed in close proximity. The film to be printed can be a solid or a liquid film. This laser-assisted printing process can also be used for the transfer of transparent materials by adding a thin layer of absorptive material (dynamic release layer) between the substrate and the material to be printed. The LIFT technique allows deposition of a controlled amount of material (a few tens of picoliters) in solid or liquid phase with high spatial resolution and a wide range of materials. This approach has been developed for more than a decade, mainly for applications in microelectronics [156,157,158,159,160,161]. These studies are often associated with real-time visualizations of the transfer, allowing a better understanding of the mechanisms, particularly in the case of a liquid layer. In liquid phase, the transfer is carried out by the formation of a jet on the free surface of the film. The dynamics of the jet and the associated deposition were studied over a wide range of viscosity for inks containing silver nanoparticles (10 to 100,000 mPa.s). These studies showed that the dynamics of the jet and the associated deposits were strongly dependent on the viscosity of the inks.

Recently, the LIFT process raised great interest in biology since scientists applied laser techniques to print two- or three-dimensional structures of any size of biomaterials, even living cells [162,163,164,165,166]. This technology of laser-assisted printing or more particularly laser bio-printing has shown a huge potential in modern medical practice and holds great promise for biological applications. We can mention the printing of a 2D model of human endothelial cells with high resolution, or the laser-induced printing of a 3D model of multilayer arrangements of fibroblasts and keratinocytes embedded in collagen as a simple model for skin [167,168]. The survival rate of the cells is close to 100%. Based on our knowledge of the LIFT process, we recently decided to combine this printing technique with stem cells technology to create ordered 2D or 3D biological in vitro architectures which could be relevant as therapeutic models [169,170,171]. We have thus demonstrated the possibility to create highly ordered cellular patterns of stem cells with a high resolution in terms of volume and location.

In these first tests, we used a bottom-up LIFT configuration for the ejection of liquid upward. This configuration makes it possible to avoid the oversizing of the film thickness at the donor center induced by the gravitation force and thus allows getting a more homogeneous film thickness. In a typical experiment, a picosecond (ps) laser with infrared radiation was used to avoid biological alteration properties due to ultraviolet radiation and to limit the temperature increase in the bio-ink. The laser beam is focused onto the donor substrate composed of a glass sample and covered by a 50 nm layer of titanium, on which a bio-ink film (e.g., biopolymer) is deposited. In the other side, the receiver is made from a microscope glass slide.

The laser-assisted transfer process is driven by several key parameters, such as the thickness of the absorbing layer, the rheological properties and the thickness of the bio-ink, the gap between donor and receiver or laser parameters (e.g., irradiated area, the fluence, etc.), and all are playing a crucial role on the printing process. All these parameters need to be accurately controlled to get a reproducible transfer process combined with a high spatial resolution to be able to create ordered matrices of bio-ink microdroplets embedded with living cells.

**Figure 10:**
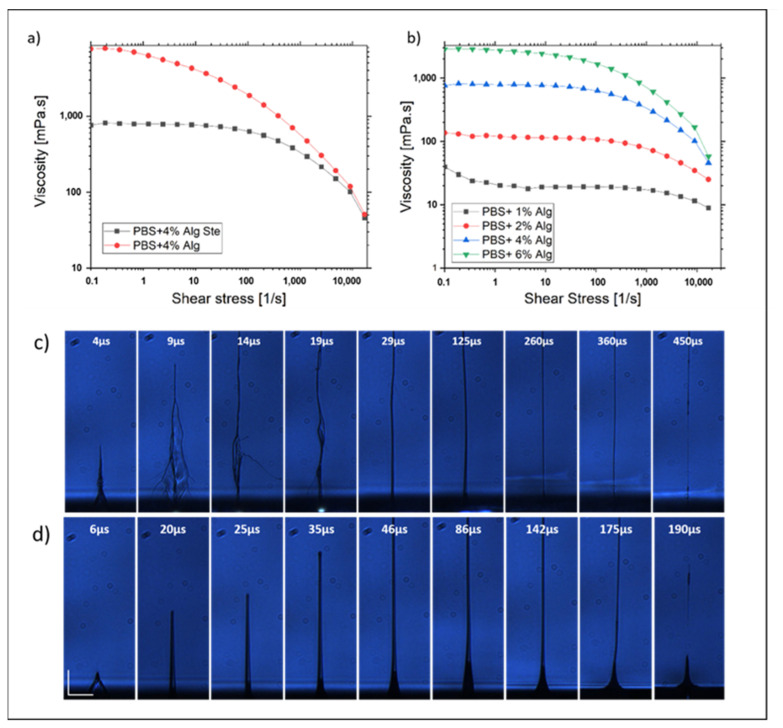
Rheological study of the bio-ink: (**a**) impact of the sterilization process on the bio-ink viscosity, (**b**) impact of the alginate percentage on the bio-ink viscosity. Jetting dynamic study by real-time visualizations for an energy of 17 µJ (**c**) 2% alg PBS bio-ink and (**d**) 4% alg PBS bio-ink (scale bar: 40 µm).

For instance, we thus noticed that a variation of a few percent in the bio-ink thickness induces a strong modification of the printing results. To improve the layer homogeneity, the surface of the donor substrate was firstly treated by plasma in order to increase its hydrophilicity before the application of the film of bio-ink. The bio-ink composition is also a key point to guarantee a good transfer result. Indeed, the rheological properties of the film, namely its viscosity, strongly impact the ejection dynamic. In addition, the bio-ink viscosity plays a major role in the spreading of the thin film and its homogeneity. For instance, to determine the best bio-ink properties, several solutions composed of phosphate-buffered saline (PBS) supplemented with 1%, 2%, 4% and 6% of alginate powder were studied. In order to be mixed with cells, the solutions have been submitted to a sterilization process (30 min, hot water vapor). Firstly, we noticed a strong decrease of the bio-ink viscosity after the sterilization process, which highlights this modification for a bio-ink composed of PBS with 4% of alginate (Figure 10a). Viscosity measurements and flow curves were performed (Figure 10b). Then, the impact of the alginate percentage was studied. The flow curves are shown in Figure 10b. They illustrate the non-Newtonian and rheo-fluidifying behavior of the solutions, and as expected, at low shear stress, the bio-ink viscosity increases with the alginate percentage. Moreover, by coupling this rheological study with real-time visualization experiments, we succeeded to highlight the link between viscosity and jetting dynamic. Indeed, a double-jetting dynamic (bounce) with lateral parasitic ejections is observed for low viscosity sterilized Dulbecco’s buffered saline (DPBS) with 2% alginate (alg) while in the case of the higher viscosity film (sterilized DPBS 4% alg), we observed a single dynamic, perfectly perpendicular to the donor substrate (Figure 10c). Obtaining this kind of well-defined jetting dynamic is primordial to allow reproducible and good printing results [172].

Based on this preliminary study, several cell printing tests were performed using the previously studied bio-ink embedded with muscular progenitors (i.e., myoblasts). After 7 days in culture (37 °C and 5% CO_2_), confluent cells are collected after Trypsin treatment and resuspended in DMEM supplemented with 10% fetal bovine serum. After collection of the cell suspension, a centrifugation step (500×rpm during 5 min) followed by removing of the supernatant is used to recover the totality of the cells. Cells are then resuspended in the sterilized bio-ink (PBS supplemented with 4% Alginate).

**Figure 11:**
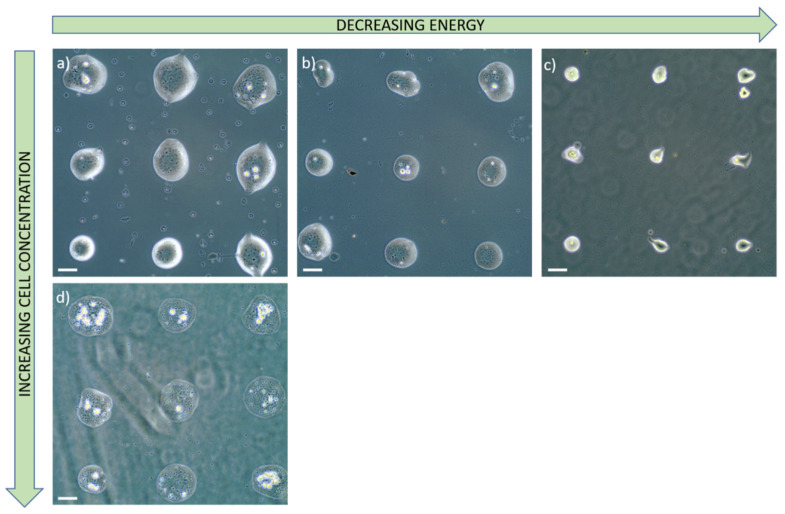
Printing results of muscular progenitors by LIFT. The laser pulse energy was 23 µJ (**a**) and (**d**), (**b**) 17 µJ and (**c**) 14 µJ. Two different cell concentrations were used: 5 million per milliliter (**a**–**c**) and 15 million per milliliter (**d**). Scale bar is 100 µm.

The working distance was set at 500 µm based on studies of jet dynamics. Under these conditions, the cells transported by the jet are deposited on the receiver with a very low velocity, which minimizes the stress to which they are subjected and prevents their degradation. By controlling the laser pulse energy and the cell concentration of the bio-ink, we proved the ability to print ordered matrices of microdroplets embedded with living cells, with different droplets size and a varying number of cells in each droplet. Figure 11 shows various results of printing with several energies by pulse (23, 17 and 14 µJ). As expected, when then energy decreases, the volume of the transferred bio-ink also decreases, allowing to reach a high ratio of single cells for an energy of 14 µJ. On the contrary, when the bio-ink cell concentration increases, for a same energy, we noticed a more important number of transferred cells (Figure 11a,d).

Laser-assisted technologies for micropatterning tissue constructs with high spatial resolution occupies a prominent place in the field of bioprinting and tissue engineering. The goal of this work was to optimize the printing parameters of the LIFT so as to transfer microdroplets of living cells with a high spatial resolution. After the bio-ink study and optimization, we showed the possibility to print ordered matrices of bio-ink droplets with embedded living cells. By controlling the laser pulse energy and the bio-ink cell concentration, we are able to control the size of each droplet and the number of transferred cells from clusters down to the single cell. These results and the possibility to print several kinds of cells in a controlled way open new perspectives for the creation of complex in vitro models combining different types of cells, such as innervated muscle fibers forming active neuromuscular junctions (by printing both muscle and motor neurons progenitors) or blood vessels (by printing endothelial stem cells). These models open future prospects for modelling a number of diseases, such as inherited neuromuscular disorder or ischemic scleroderma, among many others.

### 4.2. Laser-Induced Forward Transfer by Double Pulse (DP-LIFT): Liquid Nanojets from Solid Donors

Although the basic physical phenomena that initiate the LIFT process are photo-thermal and mechanical in nature, they are not completely understood due to the numerous effects taking place during the irradiation process. Several groups have extensively investigated the LIFT-related jetting phenomenon from low-viscosity liquid donors, both experimentally and numerically, pointing out that the jetting mechanism is initiated by an expanding cavitation bubble generated between the donor substrate and the liquid-free surface.

**Figure 12:**
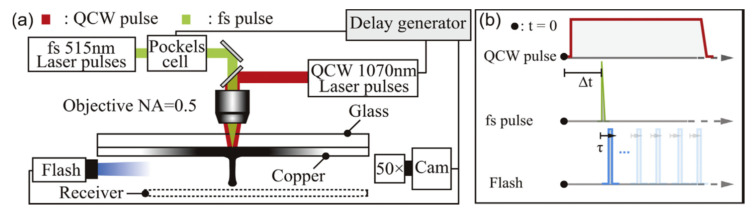
Schematic of the experimental setup for DP-LIFT. The femtosecond LIFT laser beam is represented in green and the quasi-continuous wave laser beam in red. Illumination flashlight is shown by the blue color (**a**). In (**b**), a time chart of events for synchronization of the DP-LIFT and time-resolved observations. Adapted from Reference [173].

Recently, in order to solve the limitations of single-pulse LIFT, the double-pulse laser-induced forward transfer (DP-LIFT) process has been successfully applied at the LP3 laboratory, for generating liquid nano-jets from solid donor films and, consequently, transfer of various materials (Cu, Au, Ag, Ni) on receiving substrates with well-defined and debris-free depositions. A schematic of the experimental setup for DP-LIFT is presented in Figure 12. The femtosecond LIFT laser beam is represented in green and the quasi-continuous wave laser beam in red, while illumination flashlight is shown by the blue color. A time chart of events for synchronization of the DP-LIFT and time-resolved observations is also presented. In this process, a first long-pulse laser irradiation creates a melted metal pool, and a second ultrashort pulse induces the fluid motion and initiates the jetting transfer. The influence of double-pulse parameters on the jetting phenomena has been carefully studied by means of various observation methods. To exemplify, from a fixed thickness of the donor film, debris-free single droplets with diameters ranging from 670 nm to 6.0 μm can be printed with high reproducibility. Furthermore, pillars can also be printed (as a matrix), for the fabrication of 3D microstructures. This shows the potential of this DP-LIFT approach for the development of a true-3D laser nano-/micro-printing technology that answers the need of printing materials in a serial manner following a pattern previously recorded as a digital file, much more adapted to the requirements of the digital paradigm.

To sum up, the scientific challenge in LIFT is therefore related to the intensity distribution and properties of the laser beam (pulse duration, wavelength, repetition rate), as well as the ambient medium between the donor and receiver (air, low vacuum, or a special/inert gas) and the control of the laser–matter interaction phenomena (physical and chemical transformations). Many of the technical and scientific problems have already been tackled, yet others have not been resolved, i.e., quality and versatility/universality of printing, knowledge and control of the mechanisms governing them, the insufficient resolution, as well as the high process efficiency of the technique.

### 4.3. Laser-Assisted Surface Structuring and Colloidal Lithography: A Multi-Level Multi-Physics Approach

The great success of LIFT for fluid droplets encouraged researchers in the field to also look for other ways to transfer droplets of molten solids. One way is to associate various techniques, such as by using a monolayer of transparent microspheres (e.g., by colloidal lithography, as previously presented in Section 3.2) covered with a thin metal film, and therefore to use the spheres as micro-lenses. This allows one to reach (below) diffraction limit resolution through photonic nano-jet formation, and therefore achieving a functional, versatile result. When backlit by using a single laser pulse, the laser’s interaction with the near-field mask of the microspheres produces periodic local film detachments that may be collected on a receiving substrate, leading to the printing of nanodroplets and the formation of nano-pinholes of controlled size on the microspheres. Nonetheless, when decreasing the scale to print at sub-micron and/or nanometer level (e.g., nanodroplets by microsphere-assisted LIFT), the parameters of this technique are rather difficult to control.

Several approaches have been envisaged, including microsphere-assisted laser processing, that allow the parallel ablation and printing of metal, leading to the formation of negative patterning (nano-pinholes) and positive patterning (nanodroplets) [127]. Therefore, by taking the benefit of microsphere photonic nano-jet formation, the LIFT at the nanometer scale was demonstrated for silver, and these results have been on the cover of the journal “Applied Surface Science”, volume 336 (May 2015) [127]. However, the narrow-size-distribution nanodroplets are randomly spread on the receiving surface, as observed by atomic force microscopy, revealing some of the challenges associated to downscaling to the nm-level of this laser printing technique. By producing such nanomaterials and nanostructures, one may access new, attractive optical, thermal and mechanical properties and functionalities. Such nanostructures (including arrays of pillars, dots, pinholes, hollow nanotriangles, etc.) have been created by means of laser processing, with potential use in the field of energy to enhance the absorption of light or for other surface-enhanced effects, thanks to the localized surface plasmon resonance effect.

**Figure 13:**
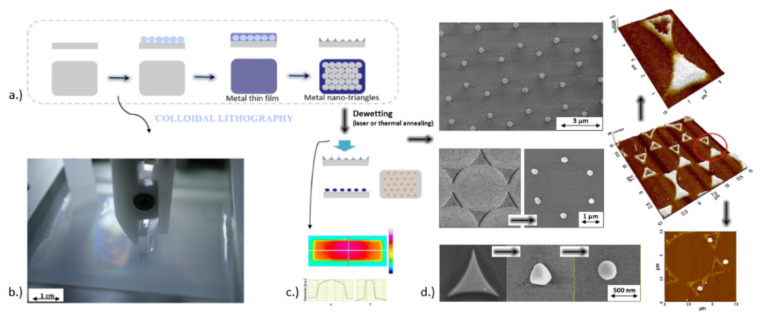
Schematic of the imagined experimental procedure of surface structuring: colloidal lithography (**a**), by using the Langmuir Blodgett technique (**b**), coupled to a controlled laser-assisted or thermal annealing de-wetting step (**c**), for various plasmonic and/or surface-enhanced Raman scattering resonators (**d**). Image partly adapted from Reference [125].

**Figure 14:**
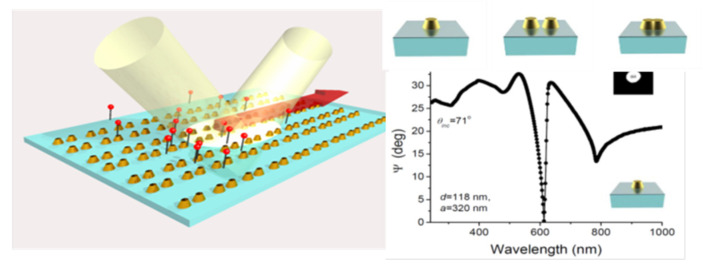
Schematic of a single molecule detection via surface lattice resonances. General presentation of measurements (left) and the surface lattice resonances in Au arrays (right). Such periodical resonating structures could be produced by means of cost-efficient LIFT and/or colloidal lithography followed by laser or thermally induced de-wetting effects, instead of the expensive e-beam lithography. Adapted from Reference [174].

An additional approach towards sub-micron and nanometer level resolution is to use colloidal lithography combined with laser processing, to obtain the surface micro-/nano-structuring with plasmon active metals [125,126,127]. Controlled laser-induced and/or thermal annealing de-wetting is a novel high-resolution yet cost-efficient technique in obtaining various structures/shapes of surface plasmon resonators (Figure 13), with the desired resolution needed in probing the structural inter-relationships of the chemical bonds. This is possible by the introduction of artificial nanostructured materials outperforming natural materials, which opens up new avenues to radically advance optical biosensing technologies. Plasmonic NPs arrays enabling surface lattice resonances (SLRs) present a prominent example of such metamaterials, providing extremely narrow, e.g., ~2–3 nm full width at half maximum, and deep resonant features, leading to singularities of light phase. Such phase singularities can be used to lower the detection limit of label-free plasmonic biosensing down to the single molecule level. A schematic for such surface structuring is presented in Figure 14, and such arrays of resonating structures could be produced by means of cost-efficient LIFT and/or colloidal lithography followed by laser or thermally induced de-wetting effects, instead of using expensive e-beam lithography [174]. The main applications are thus in close relation to artificial nanostructured materials and surfaces, in various fields of materials science, biophysics and biochemistry (tissue engineering, theranostics, laser-assisted bioprinting, scaffolding and creation of specific 2D/3D microenvironments for cell culture, etc.), but also in 3D plasmon-/2D hetero-metamaterials by surface lattice resonance-induced effects, plasmonic biosensing down to single molecule level by surface-enhanced Raman scattering, 2D layered binary metals as catalysts to split water (hydrogen generation), etc.

## 5. Laser-Induced Breakdown Spectroscopy (LIBS)

The transformation of matter into a hot plasma in the focus of an intense laser beam succeeded interest for analytical purposes immediately after the discovery of the laser [175]. However, the intense development of LIBS started much later, when reliable gain-switched pulsed laser sources became available and intensified charge-coupled devices enabled time-gated spectra recording. In the last two decades, innumerable applications have been developed in various domains [176,177,178,179]. Given the immense success of LIBS analysis on Mars [180], the technique was chosen by the American, Chinese and European space agencies for new exploration missions on the red planet starting in 2021 and 2023. In the present paper, we focus on recent developments of two particularly promising LIBS topics.

### 5.1. Fast and Sensitive Elemental Analysis of Tiny Volumes via Calibration-Free LIBS

**Figure 15:**
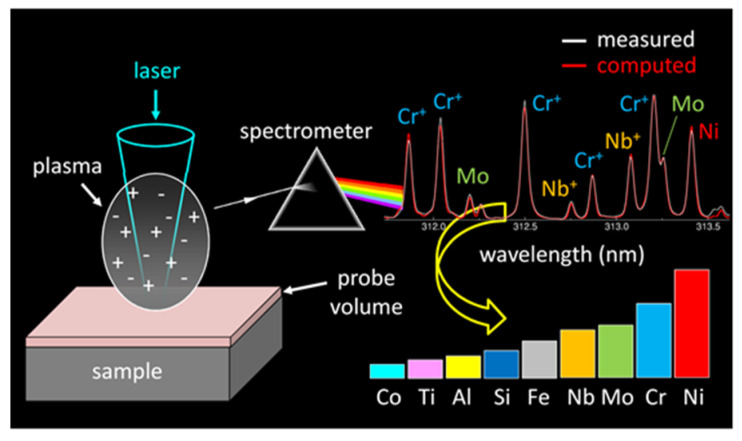
Schematic of elemental analysis via calibration-free Laser-Induced Breakdown Spectroscopy (LIBS).

Material analyses via LIBS were mostly motivated by the possibility of performing rapid standoff analysis without any sample preparation [181]. The technique however has another capability that makes LIBS unique among the analytical methods: it enables compositional measurements without any requirement of calibration with standard samples [182,183,184]. This is possible because the properties of the laser-produced plasma provide the accurate simulation of its emission spectrum using a simple and robust model (Figure 15) [185,186]. First of all, irradiation with short laser pulses (a few ns or shorter) with a fluence of the order of 100 J/cm^2^ ensures stoichiometric mass transfer from the solid sample to the plasma [187,188]. Laser ablation is a non-equilibrium process, that strongly differs from thermal evaporation that can be observed for irradiation with low fluence [189]. When the laser pulse duration exceeds the time of electron-lattice thermalization, vaporization occurs during irradiation. The laser heats the expanding vapor, thus increasing its degrees of atomization and excitation [190]. As a result of the large plume density, collisional processes dominate the radiative processes, and the plasma reaches the state of local thermodynamic equilibrium quickly after the laser pulse [191]. The plume expands rapidly during some tens of nanoseconds until a pressure equilibrium is reached between the vapor and the ambient gas [192]. Afterwards, further expansion occurs more slowly through the processes of heat and particle diffusion [193,194]. When laser ablation occurs in an inert gas such as argon, the exchange of energy between the ablation plume and the surrounding atmosphere is minimized. The plasma is therefore brighter and has a longer lifetime compared to the one produced in ambient air [195]. Moreover, it is characterized by spatially uniform distributions of temperature and densities [196]. A spatially uniform plasma in local thermodynamic equilibrium (LTE) can be considered as an ideal radiation source, because its emission spectrum can be calculated easily and accurately. Usually, both properties, the spatial uniformity and the state of equilibrium, are hardly achieved together, since the characteristic times of thermalization and diffusion are similar for atmospheric plasmas [197]. The laser-produced plasma presents an exception to this rule due to its high initial density. In fact, when the density increases, thermalization becomes faster, whereas the diffusion processes become slower [196].

**Figure 16:**
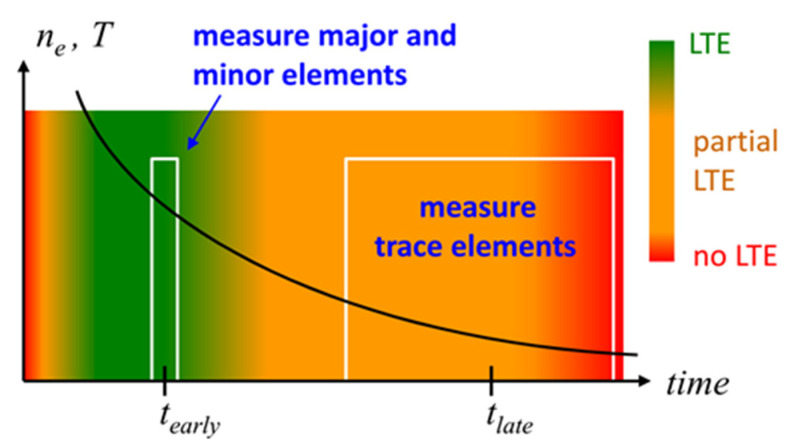
Time scheme for sensitivity-improved calibration-free LIBS: two spectra are recorded at different times. Probing the plasma in conditions of full LTE (*t_early_*) and partial LTE (*t_late_*) serves to quantify major, minor and trace elements, respectively. The color scale from green to red indicates the degree of equilibrium. Adapted from Reference [198].

Calibration-free LIBS usually suffers low sensitivity. The LTE condition of high collision rates requires large densities of charged particles and the associated continuum radiation hinders the observation of weak line emission from trace elements. Recently, we proposed an approach to overcome this drawback [198]. Considering the dependence of LTE validity on the atomic structure, we can distinguish between elements that “easily” or “hardly” reach the equilibrium state [199]. Atoms such as C, H, N and O belong to the latter group as they have large gaps of energy between their electronic excitation levels. The proposed method is therefore of particular interest for organic materials in which the “difficult” elements present the major constituents. The method is operated by measuring two spectra with different delays between the laser pulse and the detection gate, as shown in Figure 16.

**Figure 17:**
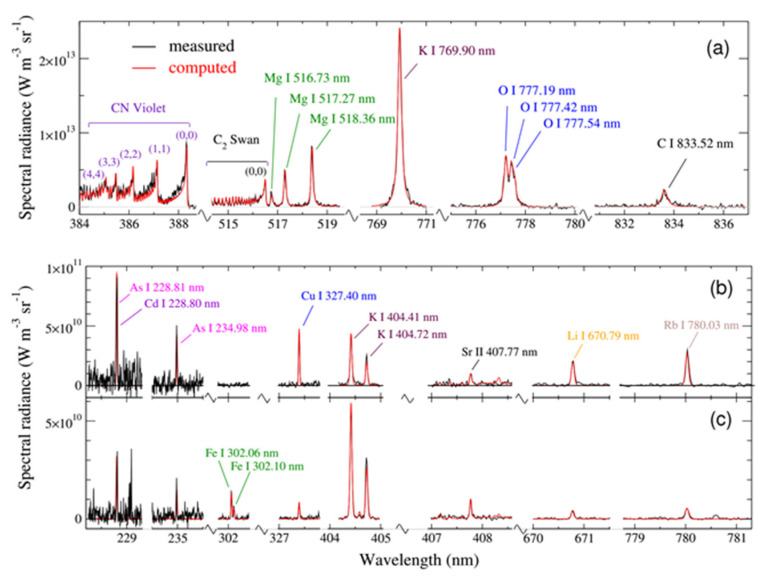
Elemental analysis of seafood. The spectrum recorded for sepia with *t* = 350 ns is (**a**) used to quantify the organic matrix and the most abundant minerals. The spectra recorded for sepia (**b**) and sardine (**c**) with *t* = 4.5 µs provide the quantification of trace elements.

The early spectrum is recorded for a time when the electron density is large enough so that all elements are in equilibrium [200]. This spectrum enables the quantification of major and minor elements. The late spectrum is recorded when the electron density is decreased, and the reduced continuum emission enables the observation of weak lines from trace elements. The state of partial LTE provides the quantification of minor and metal trace elements. The method was applied to quantify trace elements in seafood caught in the bay of Marseille [198]. The presence of arsenic (a few tens of ppm) and cadmium (ppm level) was attributed to industrial pollution (Figure 17).

**Figure 18:**
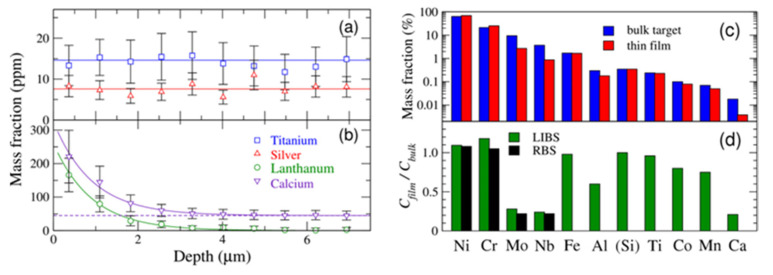
(**a**,**b**) Depth-resolved analysis of optical glass: evidence of surface contamination due to polishing [201]. (**c**) Analysis of a thin alloy film, deposited by pulsed laser deposition, and the bulk target. (**d**) Evidence of non-stoichiometric mass transfer from the target to the film. Adapted from Reference [202].

Calibration-free LIBS is also a powerful tool for spatially resolved compositional measurements. As an example, depth-resolved analysis evidenced the surface contamination of heavy flint glass due to polishing [201]. These measurements were performed by recording spectra on successive laser pulses applied to the same irradiation sites. Trace elements of the glass matrix are shown in Figure 18a to have depth-independent abundance, whereas traces of the polishing agent are located in the near-surface volume only (Figure 18b). Calcium originates from both the polishing water solution and the bulk glass, as illustrated by the exponential decrease with depth and constant value in the bulk. The bulk values equal the mass fractions measured via inductively coupled atomic emission spectrometry [201].

The analytical techniques based on calibration with standard samples are not applicable to thin film analysis and specific techniques such as Rutherford backscattering spectrometry or X-ray photoelectron spectrometry have been developed to measure the elemental composition of submicron-thick deposited layers. We have shown recently that calibration-free LIBS measurements provide analysis of multielement thin films with an analytical performance better than those obtained with other techniques [202]. The demonstration was given for a 150 nm thick nickel−chromium−molybdenum alloy thin film that was deposited by pulsed laser deposition. Analyzing the bulk target and the deposited layer, a non-stoichiometric mass transfer was evidenced (Figure 15c,d).

The main difficulty nowadays that hinders the intense development of material analysis via calibration-free LIBS is the lack of reliable spectroscopic data. Indeed, transition probabilities and Stark broadening parameters are missing or given with low accuracy for many atomic and ionic lines. The situation is however progressively improving, as illustrated by the regular updates of the widely used NIST database [203] and the numerous recent reports of new data [204,205,206].

### 5.2. LIBS Biological Imaging

**Figure 19:**
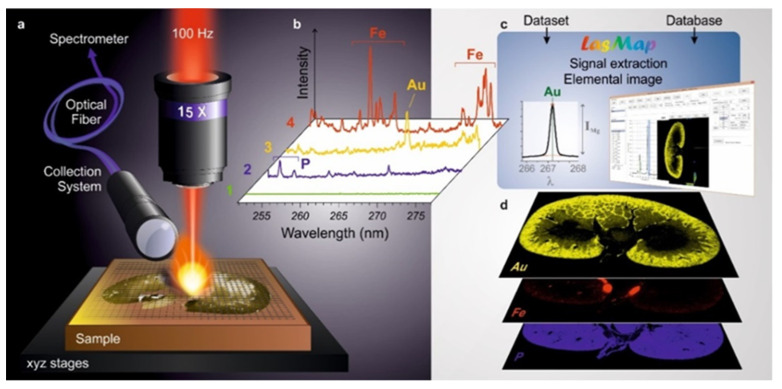
Principle of LIBS-based imaging extracted from Reference [216]. (**a**) Schematic view of a micro-LIBS configuration showing a x15 microscope lens used to focus the laser pulse, the motorized platform supporting the sample and an optical detection system connected to the spectrometer through an optical fiber. (**b**) Example of single-shot emission spectra recorded in different positions of the sample (i.e., here, a rat kidney sampled 1 h after gold nanoparticles administration) with characteristic emission lines of phosphorous (P), iron (Fe) and gold (Au). (**c**) Principle of data extraction using appropriate software. (**d**) Example of LIBS elemental images of Au (yellow), Fe (red) and P (blue).

In the past few years, the LIBS application to microscopic elemental imaging has undergone developments both in terms of instrumentation and methodology [62,207,208]. In LIBS imaging, laser-induced plasmas are generated at different positions of the sample surface with a predefined sequence covering the area of interest. The elemental information (atomic and/or ionic emission lines) is then extracted from each individual spectrum, and elemental images are obtained in a pixel-by-pixel manner, as shown in Figure 19 [209,210].

This elemental imaging approach has several advantages, including an all-optical design, a table-top instrumentation, operation in ambient atmosphere and fast operating speed (i.e., essentially related to the laser pulse repetition frequency). In addition, this technique is associated with interesting analytical performances, such as multi-element detection, detection of light elements, limits of detection in the range of ppm for most of the elements and microscopic-scale resolution [62]. It also has quantitative capabilities through the use of reference samples or various calibration strategies [209,210,211,212]. All these advantages make LIBS imaging very promising with the potential to become a reference technique in the panel of space-resolved elemental approaches, also including electron probe micro-analysis, laser ablation inductively coupled plasma mass spectrometry and synchrotron radiation microanalysis [213,214,215].

In LIBS imaging, the accessible performances (i.e., lateral resolution and limits of detection) are directly related to the laser ablation process. The best shot-to-shot repeatability is obtained when the experiment is set up in order to avoid any overlap between consecutive laser shots. The spatial resolution is then ultimately governed by the size of the craters, while the accessible limits of detection depend on both the mass of vaporized material (i.e., crater volume) and the excitation capability of the laser pulse. There is therefore a compromise to make between sensitivity and resolution, and we may note that limits of detection at the ppm scale can be typically reached for a resolution of about 10 µm. In addition, it is worth to mention that laser ablation is a micro-explosion. It is accompanied by different mechanisms, such as thermal diffusion and shock wave formation, which may cause more damage than the ablation itself. The accessible resolution therefore depends on the material properties, and in particular the hardness. However, a resolution of about 20 µm, or lower, can be achieved for a large majority of materials, including biological tissues.

The applications of LIBS-based imaging are extremely varied and cover, for example, the fields of material design [212], heterogeneous catalysis [217], paleoclimate [210], discovery of strategic metals [218,219] and preclinical studies of drugs based on metallic nanoparticles [217,218,219,220]. We describe here clinical applications of LIBS imaging to illustrate the capabilities of the technique.

The physiological and pathological roles of exogenous and endogenous metals are of major interest for the medical community [221,222,223]. High-sensitivity elemental imaging of biological tissues is however still a technological challenge that requires, in general, the use of complex instruments with restricted accesses, such as synchrotron facilities. In this sense, the LIBS performances (~20 µm resolution, ppm-scale detection limits and 100 Hz acquisition rate), its table-top instrumentation as well as its compatibility with optical microscopy make this approach very attractive for clinicians. In healthcare infrastructures, the possibility of coupling LIBS imaging with conventional histopathology characterization could be highly valuable for the completion of medical diagnoses, since it may provide important support for uncovering the elemental composition of tissues, particularly in the case of numerous pathologies related to metal exposure (respiratory diseases, dermatological diseases, cancers, etc.).

**Figure 20:**
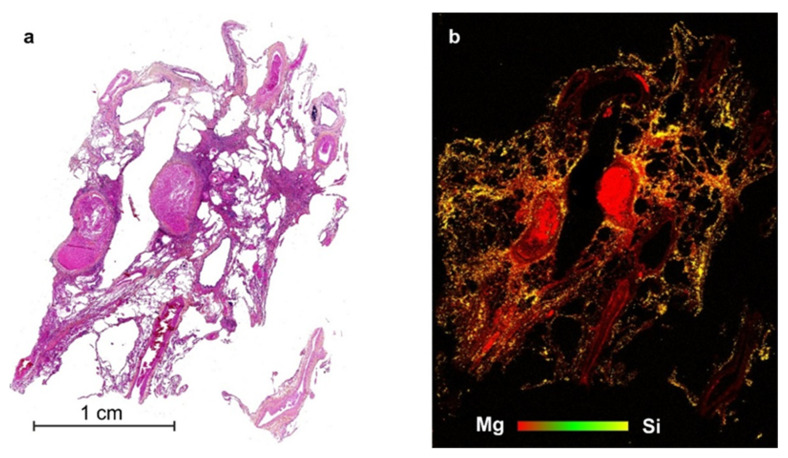
Example of human lung sample analysis, extracted from Reference [227], obtained from a patient who underwent lung transplantation for emphysema. (**a**) Histological image of the lung. (**b**) Corresponding LIBS multi-elemental images of Si and Mg. In this sample, Mg (red pixels) is used as an internal control representing the tissue. This picture shows the very high concentration of silica (yellow pixels), in the lung tissue, in a patient who, during her past occupational history, performed sandblasting during 1 year in very poor conditions.

During the last years, we have been working in collaboration with the Grenoble Alpes Hospital on the development of LIBS imaging for clinical investigations. We succeeded in adapting our methodology to human biopsies, with studies performed on normal skin and different skin cancers [224] but also on cutaneous granulomas, pigmented lymph nodes and skin scars [225]. Currently, many efforts are being made to evaluate the feasibility of LIBS imaging for pulmonary diseases. Indeed, the occupational or environmental inhalation of some metallic particles is known to cause various lung diseases such as silicosis, asbestosis, berylliosis, siderosis, hard-metal lung disease, etc. We already performed LIBS multi-elemental analysis on several dozens of human lung biopsies, after obtaining signed informed consent from every single patient. We identified several exogenous elements in the lungs of the patients (e.g., Be, Ti, Si, Al, Cr), in different lung areas and with various concentration ranges. For example, LIBS imaging led to reclassify a mislabeled idiopathic lung disease into a compensated occupational disease. Some of the results are shown in Figure 20.

Importantly, LIBS produced valuable clinical data for most of the patients and elemental analytical results were generally in accordance with patients’ past exposures. Based on these important positive preliminary findings, we initiated the first national multicenter retrospective clinical trial to evaluate the feasibility of implementing LIBS imaging as a routine diagnostic test for respiratory diseases [226]. This clinical trial involves the participation of several university hospitals in France, and 100 patients have been recruited. The active recruitment of patients is ongoing, and to date, one third of the total cohort has already been analyzed in LIBS.

The future will tell if this method may be generalized and used outside academic laboratories. In the meantime, there are remaining challenges. In particular, the analysis of complex materials (i.e., composed of several matrices) appears challenging both for quantification and data processing. Further studies dedicated to these issues will need to be conducted and the use of calibration-free LIBS and advanced chemometric tools should then offer interesting opportunities.

## 6. Conclusions

In this review, based on illustrative examples, we have highlighted the great engineering potential of laser processing approaches, so as to design varieties of materials and structures for healthcare and wellbeing challenges applications, including elemental analysis and bio-analysis. Depending on laser pulse energy, repetition frequency, focusing geometry and environmental conditions, laser-based technologies have become an alternative technological route to address a multitude of “real world” issues. For instance, the laser–matter interaction in ultraclean liquid enables to design a wealth of functional NPs exempt of any contaminants, while the “bare” NPs surface can exhibit high reactivity and much better biocompatibility compared to analogue NPs formulations made from conventional synthesis methods. Moreover, the possibility to write on the surface or inside the material at micro- and nano-scales offers great opportunities to achieve significant advances in the elaboration of novel bio-inspired functional platforms for tissue engineering applications, including calcium phosphate bioactive apatite. In addition, the possibility to transfer high energy in an ultrashort laser pulse to the matter makes the deposition of numerous materials possible, including biological ones (e.g., cells, DNA, proteins, etc.), at high resolution. The possibility confers to laser technology a promising role towards 3D bioprinting in the reconstruction of damaged native tissues or for the elaboration of complex in vitro environment for regenerative medicine. In the end, the generation of a plasma during laser–matter interaction offers precious analytical information, which can be exploited to perform rapid standoff analysis without any sample preparation in a large range of fields, including the clinical domain.

As an open question, laser-based technology in healthcare and wellbeing fields can legitimately raise interrogations about how such approaches can play a central role in contemporary worldwide issues, including climate change, transportation, energy and the very recently emerging novel virus.
